# Perspectives of substance use disorder counselors on the benefits and drawbacks of medications for opioid use disorder

**DOI:** 10.1186/s13722-025-00537-2

**Published:** 2025-02-04

**Authors:** Nicholas C. Cardamone, Rebecca E. Stewart, Kyle M. Kampman, Steven C. Marcus

**Affiliations:** 1 Department of Psychiatry, University of Pennsylvania, Perelman School of Medicine, Philadelphia, PA USA; 2 School of Social Policy and Practice, University of Pennsylvania, Philadelphia, PA USA

**Keywords:** Medications for opioid use disorder, Opioid use disorder, Substance use disorder counselors, Stigma, MOUD

## Abstract

**Background:**

Medications for opioid use disorder (MOUD) are among the best tools available to combat the opioid epidemic. Yet, use of MOUD among people with opioid use disorder (OUD) remains low. Interventions to increase MOUD access in the United States have largely focused on improving organizational capacity and addressing funding barriers, yet stigma toward MOUD may inhibit uptake even where MOUD is readily available. Non-prescribing substance use disorder (SUD) treatment professionals (e.g. counselors) likely have considerable influence on a client’s choice to initiate and adhere to MOUD, but beliefs that counselors convey about MOUD in interaction with clients are understudied. The current study explores what advantages and disadvantages that counselors communicate about buprenorphine, methadone, and naltrexone.

**Methods:**

From June to December 2021, we surveyed counselors from publicly-funded SUD treatment agencies under a municipality-wide mandate to offer MOUD to all clients with OUD. Counselors were asked to describe, in a free-response format, the most important advantages and disadvantages to communicate to their clients about taking buprenorphine, methadone, and naltrexone. Counselor responses were coded for one or more advantage and disadvantage.

**Results:**

A total of 271 SUD counselors from 29 agencies in the Philadelphia Metropolitan Area completed the survey, generating 1,995 advantages and disadvantages across three types of MOUD. The most frequently reported advantage across all three types of MOUD was their ability to reduce cravings and illicit drug use. The most frequently reported disadvantage related to the potential for some types of MOUD to develop long-term medication dependence.

**Conclusions:**

As the availability and variety of MOUD treatment options continue to expand, it is important that SUD counselors are equipped with evidence-based recommendations for OUD care. We identified misalignments with the MOUD-prescribing evidence base and stigmatizing language toward MOUD within counselors’ responses, highlighting the potential to refine training materials for MOUD and mitigate stigmatizing beliefs.

**Supplementary Information:**

The online version contains supplementary material available at 10.1186/s13722-025-00537-2.

## Background

Medications for opioid use disorder (MOUD) including buprenorphine, methadone, and naltrexone are effective at reducing both opioid related and all-cause mortality [1]. Historically, the availability of MOUD has been limited by stringent regulations on treatment agencies. However, with the state-by-state removal of prior authorization requirements, culminating in the January 2023 federal removal, MOUD treatment capacity has increased in recent years [2, 3] and a number of efforts to increase buprenorphine capacity have shown success in certain local and state contexts [4–7]. As of 2020, an estimated 42% of the U.S. population live within a 10-mile radius of a treatment facility that offers the three main types of MOUD [8]. Although more available than ever, less than a third of people with OUD or active opioid use reported receiving MOUD in the past year [9] and many individuals discontinue MOUD within a few weeks of starting [10].

Stereotypes and stigma pervade the OUD treatment system and are considerable barriers to treatment engagement, quality care, and long-term health outcomes for people with OUD [11]. A form of stigma specific to the idea of using MOUD, called “intervention stigma” [12], has been documented across the OUD treatment continuum, including in reports from community pharmacists [13], counselors and peer recovery coaches in substance use disorder (SUD) treatment facilities [14, 15], SUD treatment agency directors [16, 17], and OUD patients themselves [18]. MOUD stigma can present in different ways, such as via claims that MOUD encourages undesirable behaviors, is harmful to one’s physical or mental health, or is incompatible with “true” recovery [19].

Understanding how MOUD non-prescribing professionals, such as counselors, treat people with SUD is critical, as these professionals significantly influence client outcomes through their frequent interactions and guidance. Counselors often serve as the primary point of contact for clients, shaping their perceptions of and access to MOUD [20, 21]. Despite their pivotal role in helping people with OUD navigate recovery, little is known about how counselors discuss MOUD during client interactions [12]. To address this gap, we surveyed counselors from SUD treatment agencies in the Philadelphia Metropolitan Area to explore how they communicate the advantages and disadvantages of buprenorphine, methadone, and naltrexone to their clients. These findings aim to inform efforts to reduce stigma and align counselor communication with the MOUD-prescribing evidence base, ultimately improving MOUD access and uptake.

## Methods

### Procedures

From June to December 2021, we surveyed staff of treatment agencies that serve Medicaid recipients with SUD in the Philadelphia Metropolitan Area. These treatment agencies are reimbursed by Community Behavioral Health (CBH), the largest Medicaid payer for behavioral health in Philadelphia, and are mandated to provide MOUD to their clients with OUD [22]. In collaboration with CBH, we solicited participation from executive directors of (*n* = 49) agencies in the CBH network to distribute the survey to counselors, therapists, or any clinician who provides psychosocial support to individuals with OUD. Counselors were provided informed consent and completed the survey instrument online via REDCap, a secure, HIPAA-compliant web-based application for data collection and storage [23]. Respondents were sent a $25 Amazon gift card upon survey completion. Procedures were approved by the City of Philadelphia’s Institutional Review Board and all data were deidentified prior to analysis.

### Survey

Counselors were asked, in a free-response format, to describe the most important advantage and disadvantage they discuss with clients for each of the three MOUD, buprenorphine, methadone, and naltrexone. The prompt was designed for clarity, asking, “When discussing [medication name] with your client, what would be the most important advantage and disadvantage to mention?” After completing the survey, counselors completed a demographic questionnaire (e.g. age, gender, ethnicity, education, years of experience, etc.). Agency directors provided information about the availability of each of the three medications at their agency [17].

### Analysis

We employed thematic analysis methods [24] to code the advantages and disadvantages counselors reported for each medication. Two members of the authorship team (Author Initials, Author Initials) first collaboratively coded responses, resolving conflicts through discussion until satisfactory thematic saturation and agreement was reached. Missing responses (i.e. “I don’t know”, “not applicable”, “unintelligible”) were removed from the agreement analysis. The remaining responses were then independently coded and grouped, returning a kappa of 0.78 and 0.75 for advantages and disadvantages, respectively, indicating high inter-rater agreement [25].

## Results

### Sample characteristics

A total of 271 counselors completed the survey from 29 (59.1% agency response rate) distinct agencies who serve Medicaid recipients in Philadelphia. Five counselors could not be linked to an agency. Respondents were mostly identified as female, White, and had completed a bachelor’s degree (Table 1). The typical respondent had over a decade of professional experience and close to five years of experience at their current agency. Most respondents worked for agencies that dispensed or prescribed buprenorphine and naltrexone whereas only about half worked for agencies prescribe or dispensed methadone.

**Table 1:** Characteristics of participating counselors

Characteristic	M (SD)
Age	43.5 (13.5)
Number of years of working experience	10.7 (9.7)
Number of years in current position	4.6 (5.7)
Gender:	% (N)
Woman	192 (70.8%)
Man	73 (26.9%)
Not Reported	6 (2.2%)
Non-binary	0 (0%)
Education:	
Bachelor’s degree or higher	238 (87.8%)
Master’s degree or higher	165 (60.8%)
Race:	
Asian/Pacific Islander	7 (2.6%)
Black	100 (36.9%)
Native American	1 (0.4%)
White	139 (51.2%)
Other	18 (6.6%)
Not Reported	6 (2.2%)
Ethnicity	
Hispanic	19 (7.0%)
Not Hispanic	249 (91.9%)
Not Reported	3 (1.1%)
Agency dispenses or prescribes medication onsite	
Buprenorphine	197 (72.7%)
Methadone	128 (47.2%)
Naltrexone Unknown	222 (82.0%) 5 (1.8%)

Note: Education is not mutually exclusive. Bachelor’s degree or higher may include individuals that have attained higher levels of education (e.g. master’s, doctorates, etc.)

### Advantages

There was a total of 1,055 reported advantages across the three types of MOUD from 250 respondents. Twenty-one counselors submitted blank responses and could not be coded for an advantage. There were 375 reported advantages from 245 respondents about buprenorphine, 335 advantages from 238 respondents about methadone, and 345 advantages from 234 respondents about naltrexone. Each advantage was coded into one of 28 themes (see Supplemental File 1).

Table 2 shows most the five most frequently reported advantages across the three types of MOUD: (1) reduces urges to use (42%), (2) reduces use of illegal opioids (37%), (3) flexibility (25%), (4) supports recovery lifestyle (20%), (5) reduces withdrawal symptoms (17%). Overall, the ability to reduce opioid cravings and use were the most frequently mentioned clinical benefits for all three types of MOUD. Other clinical effects mentioned include buprenorphine and methadone’s ability to reduce overdose risk and withdrawal symptoms, and naltrexone’s ability to block opioid receptors and prevent the sedative and euphoric effects associated of opioid use.

**Table 2:** Most prevalent advantages and illustrative excerpts

Advantages	Buprenorphine (245 responses)	*n* (%)	Methadone (238 responses)	*n* (%)	Naltrexone (234 responses)	*n* (%)	Total (250 responses)
“No advantages”	-	1 (0%)	-	7 (3%)	-	0 (0%)	7 (3%)
Reduces urges to use	*“Curbs cravings while preserving general functioning.”*	76 (31%)	*“Helps reduce cravings”*	60 (25%)	*“Helping with triggers and cravings.”*	57 (24%)	104 (42%)
Reduce use of illicit opioids/substances	*“The advantages are the patient will decrease their craving for their particular substance*, *and then possibly stop using their substance of choice.”*	53 (22%)	*“[Helps] stay away from illegal usage”*	52 (22%)	*“Can help to reduce likelihood of use after treatment… No withdrawal to come off medication.”*	38 (16%)	92 (37%)
Flexible/convenient	*“Not having to go to a clinic every day to get dosed and also being able to travel out of town for a period of time because you have your doses with you.”*	11 (4%)	*-*	0 (0%)	*“It is a once-a-month injection allowing members to have more freedom.”*	57 (24%)	62 (25%)
Supports recovery lifestyle	*“The chance to chemically trick your body enabling you the time to change habits*,* though processes and obtain clean time from the substance you are desperately trying to avoid.”*	26 (11%)	*“This form of [medication assisted treatment] could support their efforts to manage their recovery management and stay away from negative people*,* places*,* and illicit activities.”*	37 (16%)	*“Of course this can offer many patients an ability to function and live a productive life.”*	14 (6%)	51 (20%)
Reduces withdrawal symptoms	*“It reduces severe opiate withdrawal symptoms*,* such as perspiration*,* nausea*,* anxiety*,* difficulty sleeping*,* chills*,* sensitivity to the light*,* and stomach pain.”*	28 (11%)	*“Clients can manage their cravings and withdrawal symptoms with methadone use*,* been used for a long time as a substitute treatment.”*	28 (12%)	*“Activat[es] to relieve craving and withdrawal it acts as a blocker*,* preventing other opioids from having any effect.”*	9 (4%)	42 (17%)

Approximately a third of respondents mention the ability for buprenorphine and methadone to reduce the use of illicit substances, jumpstart treatment readiness, and support of a recovery lifestyle. A smaller share of counselors (20%) associated these qualities with naltrexone. Instead, counselors described the advantages of naltrexone’s monthly dosing cycle: flexibility, convenience, and less interference with daily life.

### Disadvantages

There was a total of 940 reported disadvantages across the three types of MOUD from 245 respondents. Twenty-six counselors submitted blank responses and could not be coded for an advantage. There were 329 reported disadvantages from 239 respondents about buprenorphine, 353 disadvantages from 237 respondents about methadone, and 258 disadvantages from 218 respondents about naltrexone. Each disadvantage was coded into one of 26 themes (see Supplemental File 1).

Table 3 shows most the five most frequently reported disadvantages across the three types of MOUD: (1) it creates a long-term dependency (30%), (2) has harmful side effects (28%), (3) it is inconvenient (27%), (4) misuse potential (17%), (5) and difficult medication adherence (15%). Responses related to side effects frequently appeared for all three medications, including reports of cognitive and physical mal effects after taking buprenorphine or methadone and nausea or allergic reactions after a naltrexone administration. Notably, many counselors reported the tendency for buprenorphine and methadone to cause withdrawal symptoms which make it difficult for some clients to taper off.

**Table 3:** Most prevalent disadvantages and illustrative excerpts

Disadvantages	Buprenorphine (226 responses)	*n* (%)	Methadone (223 responses)	*n* (%)	Naltrexone (202 responses)	*n* (%)	Total (245 responses)
“No disadvantages”	-	10 (4%)	-	4 (2%)	-	19 (9%)	24 (10%)
Creates a long-term dependency	*“If you stay on it for years*,* it never gets them off the dependence.”*	45 (19%)	*“Methadone is often referred to as ‘liquid handcuffs’ because clients must travel to and from the clinic daily to receive it and ‘take home doses’ are difficult to earn…”*	47 (20%)	*“A person can become dependent upon it.”*	12 (6%)	74 (30%)
Side effects	*“Sedation*,* nausea*,* vomiting*,* itching.”*	35 (15%)	*“Long-term side-effects; damages teeth”*	42 (18%)	*“Nausea*,* headache*,* dizziness”; “stomach pain”*,* “fever”*,* “allergic reactions”.*	38 (17%)	70 (28%)
Inconvenient	*“Having to see the doctor so much because it is so regulated”*	13 (5%)	*“It’s a daily commitment to go to the clinic.”*	68 (29%)	*“You CAN’T [sic] miss that appointment.”*	4 (2%)	68 (27%)
Specific misuse	*“[Buprenorphine] is a divertible medication and is frequently diverted as we are learning from our clients. This means they may not use it properly and instead use it to support their ongoing addiction to fentanyl.”*	20 (9%)	*“Patient would use other substances to intensify the effects of the medication to continue use drugs or sell their medication.”*	24 (11%)	*“You can overdose if you try to get high when using it”*	9 (4%)	42 (17%)
Medication adherence or maintenance difficult	*“The most important part is keeping up with the prescription and keeping the re-fills.”*	24 (11%)	*“Must be dosed every day and if a dose is missed*,* then the patient can withdrawal and have a lot of issues. Many patients cannot keep up with it because it does have to be dosed daily.”*	14 (6%)	*“Once [on a] starting regimen many patients don’t keep up with their monthly appointments.”*	16 (8%)	39 (16%)

The main disadvantages that counselors communicated about buprenorphine and methadone related their “addictive” properties. The words “reliance”, “dependence”, “habit”, or “crutch” frequently appeared in counselor responses when describing buprenorphine and methadone. Many counselors expressed concerns about buprenorphine or methadone developing physical dependencies and the difficulty involved with weaning off the medications. In addition, counselors often described the inconvenience, stigma, and psychological attachment involved with daily dosing at a methadone clinic. Responses which mention the methadone clinic often included phrases like, “time consuming”, “schedule”, and “commitment”.

Counselors mentioned misuse potential (incl. “abuse potential”, “selling”, “mixing with other substances”, “use to get high”) in 24% of buprenorphine responses, 18% of methadone responses, and 7% of naltrexone responses. Counselors characterized MOUD misuse as selling or diverting buprenorphine and mixing methadone with other substances to achieve euphoric effects. The disadvantages counselors offered for naltrexone were primarily related to its unique properties, including the requirement of abstinence before being induced and the possibility the blocker will fail or wear off before the next dose could be administered.

Explicit stigma directed at the concept of using MOUD as an intervention for OUD were uncommon among respondents, but not absent. Phrases like “it’s substituting one drug for another” or “it’s still a drug” appeared in 18 (8%) responses for buprenorphine, 12 (5%) responses for methadone, and two (1%) responses for naltrexone.

## Discussion

Our findings highlight how non-prescribing SUD treatment professionals, such as counselors, present MOUD to their clients, revealing a mix of evidence-based benefits and perceived drawbacks. Counselors frequently emphasized advantages like MOUD’s ability to reduce urges to use and support recovery. However, reported disadvantages—including buprenorphine’s misuse potential, methadone’s dosing burden, and naltrexone’s risk of failure due to its opioid-blocking properties—often diverged from the MOUD-prescribing evidence base [26, 27]. These inconsistencies point to a critical need for targeted education, training, and standardization to align counselors’ perceptions with evidence-based practices and ensure clients receive accurate, stigma-free information about MOUD options.

A research synthesis from the National Institute of Drugs and Alcohol (NIDA) equates the effectiveness of methadone and buprenorphine at treating OUD in most treatment contexts [28]. In line with this evidence, the counselors in our sample equally praise the effectiveness of the two agonists. Yet, many respondents also claimed that the disadvantages of buprenorphine and methadone is that they can be sold or used to “get high.” The diversion risk (i.e. using medication for anything other than its intended purpose) of opioid agonists continues to be under debate. There is little evidence to support that diverted buprenorphine is primarily used for euphoric effects — several U.S. found that people with OUD who misuse their prescribed buprenorphine reported motivations ranging from self-treatment, pain-relief, to withdrawal symptoms management [29, 30].

Few respondents mentioned the diversion potential and overdose risk of methadone. Previous research has indicated that most overdose deaths involving methadone have occurred in populations that use methadone for pain relief, not OUD treatment [31]. Nonetheless, there has been a surge of scholarly interest and concern about the diversion risk of take-home methadone doses [31, 32]. The emerging evidence indicates that overdoses involving methadone decreased (not increased) among people with OUD in the U.S. after the onset of the pandemic [33, 34].

A recent NIDA report notes that naltrexone has little-to-no diversion risk and, as expected, diversion was not commonly communicated as a disadvantage from our respondents [35]. A number of respondents mentioned barriers associated with having to detox completely before being induced on naltrexone, echoing previous reports of patient experiences on extended-release naltrexone [36]. In general, respondents had less to say about naltrexone. Responses for naltrexone had the fewest number of codes extracted and substantially lower character counts than those for methadone and buprenorphine. This may be due to lack of familiarity and under-provision of naltrexone both locally and nationally [37]. Approved by the U.S. Food and Drug Administration in 2010, naltrexone has a shorter history than buprenorphine and methadone. Some evidence suggests that naltrexone may be stigmatized less than buprenorphine and methadone due pharmacological and regulatory differences [19]. Overall, our findings align with previous qualitative work with SUD counselors and supports the idea that MOUD stigma presents differently across MOUD types [20, 38].

Only a small number of respondents reported “explicit” forms of MOUD stigma (e.g. “it’s still a one drug for another”, “it’s still a drug”). However, a considerable share of responses contained language which implied that MOUD had addictive properties, harmful effects, or didn’t count as “clean time”. These misconceptions The DSM-V states that tolerance and withdrawal in the context of “appropriate medical treatment” is not considered criteria for substance use disorder [39]. Indeed, physical dependence alone is not among the criteria for a SUD diagnosis, evidence of negative consequences associated with continued is also required. These misconceptions are worrying in light of recent work in our system which shows an association between agency MOUD utilization and the endorsement of stigmatizing beliefs toward MOUD by agency leadership [17].

Encouragingly, MOUD stigma can be acted on. A series of recent randomized controlled trials to promote MOUD uptake include components that address MOUD stigma, such as facilitation collaboration with community-based treatment settings [40], implementation coaches [41], and local change teams [42]. The variability of responses in our study across counselors from one region support the potential effectiveness of training interventions that specifically focus and standardize how MOUD is presented to clients. A number of such MOUD trainings already exist, like the Providers Clinical Support System-Medications for Opioid Use Disorders [43]– however, they have not yet been tested to see if they reduce stigma among non-prescribing substance use treatment professionals. Our results also indicate the importance of tailoring destigmatization efforts to specific treatments [12] in order to provide people with OUD with all options for effective care. Future research should focus on shared decision-making models between counselors and clients and how they might be a lever to treatment success [44].

There are several limitations to the current investigation. Surveyed counselors self-reported how they discussed the advantages and disadvantages of medications, and while we sought to capture as many distinct codes as possible, there remains a risk of not fully detecting conceptual differences. Depending on individual client circumstances, what may be an advantage for one client may be a disadvantage for another. While designed to be broad, our survey prompt did not consider person-focused care or the suitability of medications across the recovery lifespan, such as how structural supports may be helpful early in treatment but burdensome later as clients transition out of treatment. Responses may also reflect counselors’ personal beliefs about MOUD rather than what they explicitly communicate to clients. Future investigations should address these limitations through independently coded recordings of counselor-client interactions to better capture the nuances of client-centered care and ensure alignment between counselors’ beliefs and their communication with clients.

In addition, there is a risk of non-response bias in our study. There is no comprehensive or published registry of SUD counselors within our system, so our sample was derived from a convenience selection of counselors spanning as many agencies as possible. Although three reminders were sent during recruitment, we lack information about non-responding counselors, making it unclear whether those who participated differed meaningfully from those who did not. Among respondents, it’s apparent that availability and administration of buprenorphine, methadone, and naltrexone vary greatly across provider agencies. Consequently, counselors’ familiarity with specific forms of each medication (oral, sublingual, or injectable) may influence their perceived advantages and disadvantages. Furthermore, our sample was geographically limited to one large urban area, which may not reflect patterns in other regions of the United States, especially where abstinence-oriented treatment facilities are more common, and exposure to MOUD may be limited [20]. Pennsylvania licensure requirements are among the most stringent for both undergraduate and graduate-level SUD counselors and state-level differences in training requirements for substance use disorder counselors further contribute to the variability in counselor knowledge and experiences with MOUD [45, 46]. Lastly, the rapid evolution of the opioid crisis, including the proliferation of synthetic opioids and the impact of COVID-19 related disruptions on SUD treatment, may have introduced new advantages and disadvantages to buprenorphine, methadone, and naltrexone which our study was unable to capture.

Despite these limitations, we believe our findings provide valuable insights into counselor perceptions of MOUD. They may help agency leaders anticipate and address stigma when implementing MOUD programs, ultimately supporting efforts to improve client access to and outcomes with evidence-based treatment options.

## Conclusions

There have been dramatic changes to the OUD treatment landscape in the last decade and the term “MOUD” encompasses a variety of medications, each with their own pharmacological properties, drawbacks, and associated stigma. New treatment options, shifting post-pandemic treatment regulations, and changes to the street drug supply have added additional complexity. SUD treatment counselors play an important role in OUD treatment and we show that the advantages and disadvantages counselors communicate vary across buprenorphine, methadone, and naltrexone and are inconsistent with the MOUD-prescribing evidence base. Our study highlights the need for ongoing education, training, and standardization around MOUD for non-prescribing SUD treatment professionals.

## Electronic supplementary material

Below is the link to the electronic supplementary material.

Supplementary Material 1

## Data Availability

The datasets analyzed during the current study are available from the corresponding author on reasonable request.

## Abbreviations

OUD: Opioid Use Disorder
MOUD: Medications for Opioid Use Disorder
SUD: Substance Use Disorder
CBH: Community Behavioral Health
