# Supplementary material for: Perspectives of substance use disorder counselors on the benefits and drawbacks of medications for opioid use disorder
Source: Addict Sci Clin Pract. 2025 Feb 4;20:7. doi: 10.1186/s13722-025-00537-2 (PMC11792642; doi:10.1186/s13722-025-00537-2)
Supplement: Supplementary file 1 — Supplementary Material 1 [file 13722_2025_537_MOESM1_ESM.docx]

Table S1. Advantages ranked by prevalence by MOUD.

| **Rank** | **Buprenorphine** | **Methadone** | **Naltrexone** |
| --- | --- | --- | --- |
| 1 | Reduces/blocks cravings, addiction, and urges or obsession to use | Reduces/blocks cravings, addiction, and urges or obsession to use | Reduces/blocks cravings, addiction, and urges or obsession to use |
| 2 | Reduces use of illegal opioids; taper; wean off illicit substances; helps maintain sobriety, promotes abstinence; reduces risk of relapse | Reduces use of illegal opioids; taper; wean off illicit substances; helps maintain sobriety, promotes abstinence; reduces risk of relapse | Flexible; convenient; less interference with daily life |
| 3 | Reduces/blocks withdrawal symptoms; prevents people from getting sick | Supports recovery lifestyle; helps achieve/sustain recovery lifestyle; improves quality of life; keeps people on the right path; promotes structure | Reduces use of illegal opioids; taper; wean off illicit substances; helps maintain sobriety, promotes abstinence; reduces risk of relapse |
| 4 | Protects against overdose/saves lives | Reduces/blocks withdrawal symptoms; prevents people from getting sick | Coder unsure/unintelligible |
| 5 | Supports recovery lifestyle; helps achieve/sustain recovery lifestyle; improves quality of life; keeps people on the right path; promotes structure | Coder unsure/unintelligible | Blocks receptors/and or blocks the effects of substances (separately from cravings, withdrawal, high) |
| 6 | Coder unsure/unintelligible | Effective; “it works” | Protects against overdose/saves lives |
| 7 | Safe; low abuse potential | Other | Supports recovery lifestyle; helps achieve/sustain recovery lifestyle; improves quality of life; keeps people on the right path; promotes structure |
| 8 | Improves treatment capacity; jump starts recovery; helps them to focus on treatment | Protects against overdose/saves lives | Client choice; more options (or forms); can tailor to patient’s individual needs, wants, types of use |
| 9 | Effective; “it works” | "I don’t know" | Safe; low abuse potential |
| 10 | Other | Safe; low abuse potential | "I don’t know" |
| 11 | Flexible; convenient; less interference with daily life | Improves treatment capacity; jump starts recovery; helps them to focus on treatment | Effective; “it works” |
| 12 | Client choice; more options (or forms); can tailor to patient’s individual needs, wants, types of use | Relieves pain | Other |
| 13 | Accessible/available | "No advantages" | Reduces/blocks withdrawal symptoms; prevents people from getting sick |
| 14 | "I don’t know" | Research evidence | “Not an opioid” |
| 15 | Research evidence | Blocks receptors/and or blocks the effects of substances (separately from cravings, withdrawal, high) | Fewer side effects |
| 16 | Blocks receptors/and or blocks the effects of substances (separately from cravings, withdrawal, high) | Client choice; more options (or forms); can tailor to patient’s individual needs, wants, types of use | It can’t be sold or diverted easily |
| 17 | Legal/FDA approved | Legal/FDA approved | Easy to administer/dose/taper down |
| 18 | Easy to administer/dose/taper down | Easy to administer/dose/taper down | Accessible/available |
| 19 | Reduces/blocks high (i.e. euphoria/attractiveness of use) | Accessible/available | Reduces/blocks high (i.e. euphoria/attractiveness of use) |
| 20 | Relieves pain | Flexible; convenient; less interference with daily life | Less stigma than other MOUDs |
| 21 | Patients can be monitored more easily | Fewer side effects | Improves treatment capacity; jump starts recovery; helps them to focus on treatment |
| 22 | Fewer side effects | It can’t be sold or diverted easily | Legal/FDA approved |
| 23 | Good for maintenance; stable dosing | Patients can be monitored more easily | Relieves pain |
| 24 | Less stigma than other MOUDs | Good for maintenance; stable dosing | Research evidence |
| 25 | Discreet | “Not an opioid” | Patients can be monitored more easily |
| 26 | "No advantages" | Reduces/blocks high (i.e. euphoria/attractiveness of use) | Good for maintenance; stable dosing |
| 27 | It can’t be sold or diverted easily | Less stigma than other MOUDs | Discreet |
| 28 | “Not an opioid” | Discreet | "No advantages" |

Table S2. Disadvantages ranked by prevalence by MOUD.

| Rank | Buprenorphine | Methadone | Naltrexone |
| --- | --- | --- | --- |
| 1 | Crutch: potential to create a long term dependency; reliance on medication; it's addictive | Inconvenience; "you have to come to clinic daily" | Side effects, long term effects, and health risks |
| 2 | Side effects, long term effects, and health risks | Crutch: potential to create a long term dependency; reliance on medication; it's addictive | Blocker issues; continued use of opioids or alcohol |
| 3 | Diversion; street value; selling | Side effects, long term effects, and health risks | "I don’t know" |
| 4 | Medication adherence or maintenance difficult; missed doses; people have to be consistent for it to be effective; takes commitment, people stop taking it. | Specific misuse; "you can take too much"; mixing with other substances “to get high”; continued use of illicit substances concurrently | Requires detox, abstinence before starting |
| 5 | Specific misuse; "you can take too much"; mixing with other substances ”to get high”; continued use of illicit substances concurrently | Interferes with other treatment, housing, or employment | Risk of overdose or risk of relapse from the medication or misuse of the medication |
| 6 | Induces or causes withdrawal (i.e. precipitated withdrawal) | Abuse potential | Medication adherence or maintenance difficult; missed doses; people have to be consistent for it to be effective; takes commitment, people stop taking it. |
| 7 | Abuse potential | Induces or causes withdrawal (i.e. precipitated withdrawal) | Coder unsure/unintelligible |
| 8 | Ineffective; “it doesn’t work”; ”medication itself is not enough”; it won’t work due to certain caveats and without additional supports (e.g. changes in drug supply) | Medication adherence or maintenance difficult; missed doses; people have to be consistent for it to be effective; takes commitment, people stop taking it. | Other disadvantage |
| 9 | Still a drug; subbing | Coder unsure/unintelligible | Ineffective; “it doesn’t work”; ”medication itself is not enough”; it won’t work due to certain caveats and without additional supports (e.g. changes in drug supply) |
| 10 | Risk of overdose or risk of relapse from the medication or misuse of the medication | "I don’t know" | Crutch: potential to create a long term dependency; reliance on medication; it's addictive |
| 11 | Inconvenience; "you have to come to clinic daily" | Still a drug; subbing | Painful administration |
| 12 | Coder unsure/unintelligible | Stigma | Specific misuse; "you can take too much"; mixing with other substances ”to get high”; continued use of illicit substances concurrently |
| 13 | Stigma | Other disadvantage | Dosing challenges; timing of dose; complicated to start; holding dose; interactions with other medications; lapses in dosing |
| 14 | "No disadvantages" | Risk of overdose or risk of relapse from the medication or misuse of the medication | Inaccessible; difficult to find a doctor, maintain prescriber, or insurance coverage |
| 15 | Interferes with other treatment, housing, or employment | Dosing challenges; timing of dose; complicated to start; holding dose; interactions with other medications; lapses in dosing | Induces or causes withdrawal (i.e. precipitated withdrawal) |
| 16 | Other disadvantage | Ineffective; “it doesn’t work”; ”medication itself is not enough”; it won’t work due to certain caveats and without additional supports (e.g. changes in drug supply) | Expensive |
| 17 | "I don’t know" | Diversion; street value; selling | Inconvenience; "you have to come to clinic daily" |
| 18 | Inaccessible; difficult to find a doctor, maintain prescriber, or insurance coverage | "No disadvantages" | Stigma |
| 19 | Triggering to patient and others | Inaccessible; difficult to find a doctor, maintain prescriber, or insurance coverage | Abuse potential |
| 20 | Expensive | Triggering to patient and others | Interferes with other treatment, housing, or employment |
| 21 | Dosing challenges; timing of dose; complicated to start; holding dose; interactions with other medications; lapses in dosing | Requires detox, abstinence before starting | Still a drug; subbing |
| 22 | Patients can’t be monitored as easily | Less patient autonomy; fewer choices | Diversion; street value; selling |
| 23 | Requires detox, abstinence before starting | Blocker issues; continued use of opioids or alcohol; ”try and override the blocker”; ”It’s just a blocker”; ”people take more to get around it”; ”only partially blocks so you can still get high” | "No disadvantages" |
| 24 | Less patient autonomy; fewer choices | Expensive | Triggering to patient and others |
| 25 | Blocker issues; continued use of opioids or alcohol; ”try and override the blocker”; ”It’s just a blocker”; ”people take more to get around it”; ”only partially blocks so you can still get high” | Patients can’t be monitored as easily | Less patient autonomy; fewer choices |
| 26 | Painful administration | Painful administration | Patients can’t be monitored as easily |
